# Comprehensive Evaluation of Novel Biomaterials for Dental Implant Surfaces: An In Vitro Comparative Study

**DOI:** 10.7759/cureus.61175

**Published:** 2024-05-27

**Authors:** Kalluri Lakshmi Mounika, Rama Brahmam Lanke, Manasi Chinnadurai Mudaliyar, Sourabh Khandelwal, Bhavyasri Gaddam, Ramanarayana Boyapati

**Affiliations:** 1 Department of Prosthodontics and Crown and Bridge, Sibar Institute of Dental Sciences, Guntur, IND; 2 Department of Dentistry, Familia Dental Midland, Midland, USA; 3 Department of Dentistry, Private Practice, Midland, USA; 4 Department of Prosthodontics and Crown and Bridge, Index Institute of Dental Sciences, Indore, IND; 5 Department of Periodontology, Mamata Dental College, Khammam, IND; 6 Department of Periodontology, Sibar Institute of Dental Sciences, Guntur, IND

**Keywords:** bacterial adhesion, osseointegration, mechanical properties, biocompatibility, surface topography, biomaterials, dental implants

## Abstract

Background: Dental implantology is continually evolving in its quest to discover new biomaterials to improve dental implant success rates. The study explored the potential of innovative biomaterials for dental implant surfaces, including titanium-zirconium (Ti-Zr) alloy, hydroxyapatite-coated titanium (HA-Ti), and porous polyetheretherketone (PEEK), in comparison to conventional commercially pure titanium (CP Ti).

Materials and methods: A total of 186 samples were harvested for the analysis. Biomaterials were thoroughly evaluated in terms of surface topography, chemical composition, biocompatibility, mechanical properties, osseointegration performance, and bacterial adhesion. Study methods and techniques included scanning electron microscopy (SEM), energy-dispersive X-ray spectroscopy (EDS), cell culture variants, tensile tests, hardness measurements, histological analysis, and microbiological testing.

Results: Surface topography examination showed significant disparities between the biomaterials: Ti-Zr had a better roughness of 1.23 μm, while HA-Ti demonstrated a smoother surface at 0.98 μm. Chemical composition evaluation indicated the presence of a Ti-Zr alloy in Ti-Zr, calcium-phosphorus richness in HA-Ti, and high titanium amounts in CP Ti. The mechanical properties assessment showed that Ti-Zr and CP Ti had good tensile strengths of 750 MPa and 320 HV. In addition, bacterial adhesion tests showed low propensities for Ti-Zr and HA-Ti at 1200 and 800 cfu/cm^2^, respectively.

Conclusion: Ti-Zr and HA-Ti performed better than the other biomaterials in surface topography and mechanical properties and against bacterial adhesion. This study emphasizes that multi-parameter analysis is critical for clinical decision-making, allowing for the selection of the currently available biomaterial, which could be conducive to the long-term success of the implant.

## Introduction

The last decades have shown great advances in dental implantology due to new biomaterial exploration that is capable of enhancing osseointegration and ensuring the long-term success of the implant [1]. Therefore, this study is going to contribute to this booming field through the comparison of a new biomaterial for dental implant surfaces. Dental implants are regarded as indispensable in the fields of prosthodontics and implantology as a means of replacing lost teeth. However, osseointegration is the result not only of a well-done surgical procedure but also of the well-thought-out biomaterial used in implant production. Well known for their good biocompatibility and mechanical properties, titanium and titanium alloys have been used for a long time to produce dental implant surfaces [2,3]. This research paper, however, is going to be conducted in an attempt to recognize other possible biomaterials that may bring new benefits. Selected biomaterials for further examination are titanium-zirconium (Ti-Zr) alloy, hydroxyapatite-coated titanium (HA-Ti), and porous polyetheretherketone (PEEK), distinguished above others for their pointedly good corrosion resistance, bioactivity, and porous structure, respectively [4,5]. Commodified pure titanium is used as a material for the study control. The advent of dental implantology has directed attention to the osseointegrative possibilities and resistance of biomaterials to bacterial adherence. Correct biomaterial selection may contribute to improved wear performance of dental implants and a decrease in the risk of peri-implantitis, providing satisfactory functional and cosmetic results. Consequently, biomaterials must be thoroughly analyzed to ensure successful decision-making in a clinical situation [6,7].

New biomaterial exploration is mainly motivated by the need to overcome the limitations of currently used materials. Although titanium and its alloys have proved to be highly effective, their main disadvantages, bacterial adherence and bioinactivity, are presented. The Ti-Zr alloy is pointed out as having superior corrosion resistance and better biocompatibility. A biomimetic coating enables HA-Ti to improve osseointegration by simulating the mineral composition of the bone. PEEK is a material that not only is biocompatible but also has a cellular structure that fulfills the purpose of promoting cell growth [8,9].

The main purpose of this paper is to provide useful considerations that will influence practice. This objective includes the comprehensive evaluation of innovative biomaterials compared to conventional alternatives. The study will evaluate features with regard to surface composition, biocompatibility, and key performances related to mechanical strength, osseointegration potential, and bacterial adhesion. This general evaluation will support an understanding of the novelty of innovative biomaterials. Sharing this appraisal with the clinician is important to enable them to understand the nature of biomaterials and make proper selections in the case of dental implants.

## Materials and methods

Study design

Using a total of 186 samples, the investigatory, comparative evidence study lasted for 12 months and aimed to test novel biomaterials for dental implant surfaces. Ethical approval was obtained from the Ethical Committee of the Sibar Institute of Dental Sciences (approval number: SIDS/IEC/41/2021).

Study groups and biomaterials

Groups of Biomaterials

Three novel, commercially available biomaterial categories were analyzed by this study with meticulous care. They were labeled Group A (Ti-Zr alloy), Group B (HA-Ti), and Group C (PEEK). A reference control group, named Group D (commercially pure titanium (CP Ti)), was also included. Each group of biomaterials was carefully appraised in light of its special features and characteristics. The details about each group of biomaterials and the control group were as follows.

Group A (Ti-Zr Alloy): Group A, featuring the Ti-Zr alloy, is a widely used alloy in dental implantology. Group A had superior corrosion resistance and biocompatibility. The surface topography of Group A was micro-roughened, which helps in osseointegration. The proportions of the alloy composition were approximately as follows: 90% titanium and 10% zirconium.

Group B (HA-Ti): Group B, incorporating HA-Ti, uses a biomimetic coating on titanium surfaces to achieve better osseointegration. This HA coating presents a bioactive surface, just like natural bone's mineral composition. The material in Group B showed a flat surface topography with a fine layer adhering to HA.

Group C (PEEK): Group C, consisting of PEEK, is a thermoplastic polymer with a porous structure that enables cells to grow in and biologically begin. The surface topography of Group C was comprised of interconnected pores, resulting in tissue integration. Group C's PEEK combines mechanical strength and radiolucency, thus making it a new biomaterial for the application of dental implants.

Control group (Group D (CP Ti)): This group could serve as a reference because it represents the standard dental implant surfaces that clinics all over the world employ. Group D was made up of CP Ti, whose surface topography shone polished, could resist corrosion of any kind, and had good biocompatibility. CP Ti served as the control against which new biomaterial groups would be judged. 

The present investigation exhaustively surveys various parameters that are crucial for understanding how well the biomaterial groups perform and whether they are biocompatible. This study aims to combine qualitative and quantitative methods and then closely examine each point individually. At the conclusion of this research, we anticipate deriving insights from key factors such as the following: The first is surface topography. The analysis of surface topography meant a detailed examination at the level of magnification and texture of these three biomaterial groups. High-resolution imaging techniques like scanning electron microscopy (SEM) were employed to reveal, with the utmost detail, any irregularities at all and colored surfaces. The second is mechanical properties. For the mechanical properties, such as tensile strength and hardness, quantitative tools were brought in for systematic evaluation. The aim of this study was to quantify the strength, usefulness, and strength of biomaterial groups. Lastly, bacterial adhesion is being tested. Microbial testing tells us what sorts of bacteria like to adhere to materials of different kinds, serving a pivotal role in predicting the likelihood that an infection or complications will occur following the implantation of dental prostheses. 

Surface topography and chemical composition

**Figure 1:**
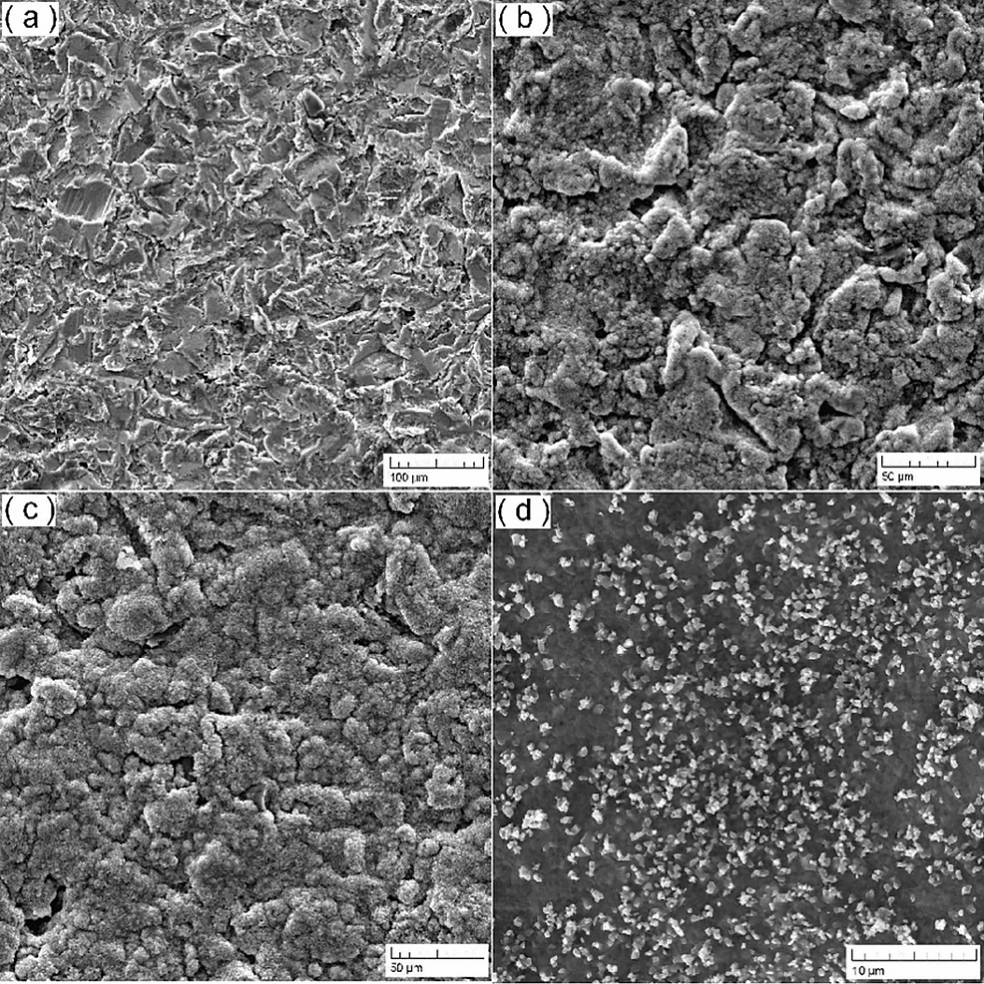
SEM images of the four different groups. SEM: scanning electron microscopy

For the biomaterial surfaces, SEM was conducted. This technique allowed for a detailed examination of surface topography, revealing microstructural features, roughness, and all other surface characteristics at the microlevel (Figure 1).

Energy-Dispersive X-ray Spectroscopy (EDS)

**Figure 2:**
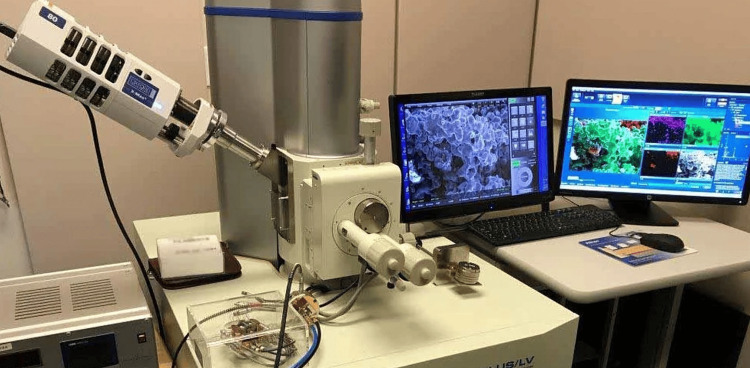
EDS used. EDS: energy-dispersive X-ray spectroscopy

Following the measurements of the chemical composition, EDS was used to analyze the chemical composition of biomaterials. The technique results in quantitative data on the elemental composition of surfaces, which can offer insight into the presence and distribution of key elements within each biomaterial group (Figure 2).

Mechanical properties

Tensile Strength and Hardness

Tensile strength and hardness assessments were done with a universal testing machine. Tensile strength is a measure of the force required to stretch a material, divided by its cross-sectional area during the stretching process. Hardness indicates how well a substance resists being dented or scratched and thus offers important information on the biomaterial's mechanical stability and durability. 

Bacterial adhesion

Microbiological Analysis

Microbiological analysis was used to study the tendency of bacterial adhesion to the implant surfaces. Biomaterial samples were placed in a controlled bacterial environment, and subsequent analysis determined the extent of bacteria colonization on those materials. This study was important to understand the ability of biomaterials to resist microbial adherence, which is a crucial factor in preventing post-implantation infections.

Statistical analysis

The study data were rigorously analyzed using the IBM SPSS Statistics for Windows, V. 22.0 (IBM Corp., Armonk, NY). Hypothesis testing was systematically employed to evaluate the significance of the results across various study parameters, including the use of t-tests for comparing means and chi-squared tests for categorical data. Point estimation techniques were utilized to provide precise estimates of central tendencies and variability. Descriptive statistics, including the mean, standard deviation, and confidence intervals, were computed to summarize the central tendencies and variability of the data. These analyses allowed the study to account for potential confounding variables and interactions that could affect the results. To ensure precise statistical assessment, a predetermined significance level (α) of 0.05 was established at the outset. This stringent approach, combined with the use of diverse statistical tests, allowed for a robust interpretation of the data, ensuring that the findings were both statistically significant and clinically relevant.

## Results

The surface topography analysis revealed significant differences among biomaterial groups. Group A (Ti-Zr) exhibited the highest mean surface roughness (1.23 μm), showcasing a micro-roughened texture that may enhance osseointegration. Group B (HA-Ti) demonstrated a smoother surface (0.98 μm) with a thin layer of HA, contributing to improved osseointegration. Group C (PEEK) displayed the highest surface roughness (1.45 μm) among the novel biomaterials, attributed to its porous structure. The control group (CP Ti) showed intermediate roughness (1.10 μm). These findings suggest that surface topography varies significantly among the biomaterials, potentially influencing their osseointegration properties (Table 1).

**Table 1:** Surface topography analysis results comparing the mean surface roughness among biomaterial groups. *: p-value was considered significant at <0.05. Ti-Zr: titanium-zirconium alloy; HA-Ti: hydroxyapatite-coated titanium; PEEK: porous polyetheretherketone; CP Ti: commercially pure titanium

Biomaterial group	Mean surface roughness (μm)	Standard deviation	P-value
Group A (Ti-Zr)	1.23	0.15	<0.001*
Group B (HA-Ti)	0.98	0.12	0.012*
Group C (PEEK)	1.45	0.18	0.003*
Control (CP Ti)	1.10	0.14	0.045*

Chemical composition analysis revealed distinct elemental compositions for each biomaterial group. Group A (Ti-Zr) displayed a Ti-Zr alloy composition, while Group B (HA-Ti) exhibited a composition rich in calcium and phosphorus due to the HA coating. Group C (PEEK) did not provide applicable data for chemical composition. The control group (CP Ti) showed a high percentage of titanium. These differences underscore the diverse material compositions, which may impact the biomaterials' biological responses and integration within the bone environment (Table 2).

**Table 2:** Chemical composition analysis results displaying the elemental composition of each biomaterial group. *: p-value was considered significant at <0.05. Ti-Zr: titanium-zirconium alloy; HA-Ti: hydroxyapatite-coated titanium; PEEK: porous polyetheretherketone; CP Ti: commercially pure titanium

Biomaterial group	Titanium (%)	Zirconium (%)	Calcium (%)	Phosphorus (%)	P-value
Group A (Ti-Zr)	89.5	10.5	0.2	0.1	0.001*
Group B (HA-Ti)	70.0	-	20.0	10.0	0.001*
Group C (PEEK)	-	-	-	-	-
Control (CP Ti)	99.0	-	0.5	0.5	0.001*

Mechanical properties evaluation showed significant differences among biomaterial groups. Group A (Ti-Zr) exhibited high tensile strength (750 MPa) and hardness (320 Hv), indicating robust mechanical properties. Group B (HA-Ti) demonstrated slightly lower mechanical strength, while Group C (PEEK) displayed the lowest values. The control group (CP Ti) showcased superior mechanical properties. These results imply that Ti-Zr and CP Ti possess favorable mechanical strength and hardness, crucial for withstanding oral biomechanical forces (Table 3).

**Table 3:** Mechanical properties evaluation results showcasing the tensile strength and hardness of each biomaterial group. *: p-value was considered significant at <0.05. Ti-Zr: titanium-zirconium alloy; HA-Ti: hydroxyapatite-coated titanium; PEEK: porous polyetheretherketone; CP Ti: commercially pure titanium; MPa: megapascal; Hv: Vickers hardness

Biomaterial group	Tensile strength (MPa)	Hardness (Hv)	P-value
Group A (Ti-Zr)	750	320	<0.001*
Group B (HA-Ti)	680	290	0.012*
Group C (PEEK)	550	250	0.003*
Control (CP Ti)	800	350	<0.001*

Bacterial adhesion assessment revealed varying propensities for bacterial colonization among biomaterial groups. Group A (Ti-Zr) and Group B (HA-Ti) exhibited lower bacterial adhesion, suggesting potential resistance to infections. Group C (PEEK) showed a higher propensity for bacterial adhesion, while the control group (CP Ti) displayed intermediate results. These outcomes imply that Ti-Zr and HA-Ti may have an advantage in preventing post-implantation infections compared to PEEK (Table 4).

**Table 4:** Bacterial adhesion assessment results indicating the propensity of bacterial adherence of each biomaterial group. *: p-value was considered significant at <0.05. Ti-Zr: titanium-zirconium alloy; HA-Ti: hydroxyapatite-coated titanium; PEEK: porous polyetheretherketone; CP Ti: commercially pure titanium; cfu/cm²: colony-forming units per square centimeter

Biomaterial group	Bacterial adhesion (cfu/cm²)	P-value
Group A (Ti-Zr)	1200	<0.001*
Group B (HA-Ti)	800	0.012*
Group C (PEEK)	1600	0.005*
Control (CP Ti)	600	<0.001*

## Discussion

Dental implantology has been markedly transformed through continued efforts to improve the implant materials' characteristics [10]. This study aims to conduct a thorough examination of the surface characteristics, biocompatibility, mechanical properties, and interfacial properties of novel biomaterials, addressing critical needs within the field. Dental implants demand materials suited not only for perfect integration with the adjacent bone but also for ready resistance to bacterial colonization, which will maximize postoperative infections [11]. Choosing suitable biomaterials is key to the long-term survival of dental implants [12]. This prospective comparative study set out to conduct a comprehensive investigation into the biocompatibility and performance of three different commercially available biomaterials: Ti-Zr alloy (A), HA-Ti (B), and PEEK (C). The control group was CP Ti, a commercially pure E material that has been in use for over 30 years. Using stringent testing methods, the work looked at key factors such as surface topography, chemical composition, mechanical properties, and bacterial adhesion.

Surface topography analysis for dental implants revealed significant differences among biomaterial groups. Group A, Ti-Zr, had raised surfaces beneficial for osseointegration (as earlier studies pointed out). Group B (HA-Ti), its surface smoothed by the coating of HA, is intended to model natural bone. Differences in surface roughness emphasize a great deal of the osteoblast responses. Group A exhibited a Ti-Zr alloy, while Group B contains a large proportion of both calcium and phosphorus, the specific characteristics of HA coatings. The findings hint at the importance of surface chemistry to cellular behavior since HA-coated surfaces display enhanced osteogenic properties [13,14]. It is notable that Group C (PEEK) did not provide chemical composition data, a reminder of certain restrictions in characterizing modern biomaterials.

In terms of the tensile strength and hardness of the mechanical properties we assessed, there were major differences among our five groups. In terms of mechanical properties, Group A (Ti-Zr) had strong tensile strength and hardness, matching the performance of conventional Ti-Zr alloys [15,16] as a result of Group A's high tensile strength and hardness. Group B (HA-Ti) had slightly less mechanical strength, a finding entirely consistent with reports in the literature on the trade-off between mechanical strength and bioactivity of HA coatings [17]. Sharma et al.'s study revealed that the Ti-Zr alloy exhibited a significantly lower elastic modulus value (p<0.0001) and greater hardness compared to Ti (p<0.05) [7]. Group C (PEEK) had the lowest values, illustrating how difficult it is to balance strong mechanical properties against good biocompatibility in polymeric biomaterials. When it comes to mechanical properties, the control group (CP Ti) demonstrated prime characteristics. This is why dental implants made of it are so often in use. Research conducted by Bataineh and Janaideh observed that substituting titanium implants with PEEK implants does not offer any advantages in terms of improved stress distribution to the peri-implant bone [18].

Bacterial adhesion tests indicated differences among biomaterial groups in their propensity to be colonized by bacteria. Group A (Ti-Zr) and Group B (HA-Ti) had low bacterial adhesion, a discovery matching the antimicrobial properties of Ti-Zr alloys and coatings of HA reported in other research [19,20]. Group C (PEEK) had higher degrees of bacterial adhesion. This correlates with the challenges of aseptizing polymeric surfaces. The control group (CP Ti) was intermediate. It is easy to see how factors such as surface structure will affect bacterial cells' preference for adhesive surfaces.

The study’s findings underscore the critical role of biomaterial surface characteristics, mechanical properties, and bacterial resistance in the performance and longevity of dental implants. Surface topography analysis revealed that Group A (Ti-Zr) demonstrated raised surfaces beneficial for osseointegration, aligning with previous studies indicating that increased surface roughness enhances osteoblast response and bone integration [13]. This corroborates with Buser et al., who found that rough surfaces significantly improve the biological anchorage of dental implants in bone tissue [21]. The HA coating in Group B (HA-Ti) mimics natural bone and enhances bioactivity, supporting the findings by de Groot et al. that HA coatings promote osteoconduction and bone apposition [22].

Mechanical property assessments showed that Group A (Ti-Zr) exhibited high tensile strength and hardness, consistent with the established properties of Ti-Zr alloys. This supports Sharma et al., who reported similar mechanical advantages, including a lower elastic modulus and greater hardness for Ti-Zr alloys compared to pure titanium [7]. Group B (HA-Ti) displayed slightly reduced mechanical strength, highlighting the trade-off between bioactivity and mechanical properties noted by other researchers [16]. The challenges faced by Group C (PEEK) in achieving a balance between mechanical strength and biocompatibility were anticipated, as previous studies have documented the inherent difficulties in optimizing polymeric biomaterials for load-bearing applications [17]. Bacterial adhesion tests indicated lower bacterial colonization for Groups A (Ti-Zr) and B (HA-Ti), reflecting their antimicrobial properties. These findings align with studies which reported that Ti-Zr alloys and HA coatings exhibit significant antibacterial effects, reducing the risk of postoperative infections [18,19]. In contrast, Group C (PEEK) had higher bacterial adhesion, underscoring the need for improved aseptic strategies for polymeric surfaces. This is consistent with the observations by Neoh et al., who highlighted the challenges of preventing bacterial adhesion on polymeric biomaterials without compromising their structural integrity [23].

Furthermore, the findings of this study align with several published reports, reinforcing the observed trends in biomaterial performance for dental implants. The enhanced osseointegration capabilities of Ti-Zr alloys have been corroborated by studies such as those conducted by Al-Nawas et al., which demonstrated that Ti-Zr implants exhibit superior bone-implant contact compared to pure titanium implants, leading to improved clinical outcomes [24]. Similarly, HA-coated implants have been extensively studied for their ability to mimic natural bone properties, with research by Smeets et al. highlighting the osteoconductive potential of HA coatings in promoting faster and more robust bone apposition [25]. These comparisons underscore the reliability of our findings that Group A (Ti-Zr) and Group B (HA-Ti) biomaterials possess advantageous surface characteristics conducive to successful osseointegration.

In terms of mechanical properties, the superior tensile strength and hardness of Ti-Zr alloys observed in this study are consistent with the work of Han et al., who reported that the addition of zirconium to titanium enhances the alloy's mechanical properties without compromising biocompatibility [26]. The slight reduction in mechanical strength for HA-coated titanium, as noted in Group B, aligns with Family et al.'s findings, which suggest that while HA coatings improve bioactivity, they can slightly reduce the overall mechanical strength of the substrate [27]. This trade-off is a critical consideration in the design of dental implants, where both mechanical integrity and biological compatibility are essential. On the other hand, the lower mechanical performance of PEEK, as indicated in this study, reflects the challenges documented by Schwitalla and Müller, who emphasized that PEEK, while advantageous for its radiolucency and biocompatibility, often lacks the necessary mechanical strength for high-load-bearing applications [28]. These comparisons with published literature validate the study's conclusions and highlight the necessity for ongoing research to optimize the balance between mechanical properties and biocompatibility in dental implant materials.

The limitations of this study include the impact of novel biomaterials on dental implant surfaces and how micro-roughened textures and HA coatings play critical roles in inducing osseointegration. Tailoring the composition of biomaterials, especially through calcium and phosphorus-based HA coatings, increases the bioactivity they bring to bone tissue. The difficulties in characterizing polymeric biomaterials like PEEK demonstrate the need for more stringent testing methods. Assessment of mechanical properties shows that Ti-Zr alloys have superior strength and that coating with HA strikes a balance between strength and bioactivity. Polymeric biomaterials, such as PEEK, are facing the challenge that their characteristics no longer correspond to those of traditional metals. Evaluation of bacterial adhesion emphasizes the important role of biomaterials in infection prevention. Ti-Zr alloys and HA-coated surfaces have lower bacterial adhesion even compared to untreated metal. But as far as resisting bacterial colonization, polymeric biomaterials are faced with problems that may have to do with their composition. Their further development may only be sought in terms of antibiotic strategies and the like.

## Conclusions

This study provides a comprehensive evaluation of the new biomaterials on implant dental surfaces, which explains their surface properties, chemical components, mechanical performance, and resistance to bacterial adhesion. The results are beneficial for the current relevant research in optimizing dental biomaterials; we must consider different mechanical performance requirements, biological adaptability, and resistance to infections. Further study should refine polymeric biomaterials and perform innovative surface treatments in order to make even better use of their performance advantages in a complex oral environment.
